# False Business Practice

**Published:** 1858-04

**Authors:** 


### FALSE BUSINESS PRACTICES.

Dentists desire and need DEPOTS in Cincinnati, devoted *exclusively* to their *interests*, conducted liberally and on the most comprehensive scale, where they can obtain everything desired,—promptly and at the *lowest* Eastern *prices*. Yet there is a large portion of the profession who act as if these were only *accommodating* mediums. When they want $25, $50, or $100 worth of goods on which there is a reasonable profit, they send their orders East; but when they want one or two teeth for a difficult case, or a piece of gold plate on which the profits scarcely pay for the trouble, they send to Cincinnati, and frequently *without money*, at that.

A few days ago we met a Dentist from a neighboring city, where a small stock of Dental Goods is kept. In conversation he remarked, "We have a House professing to keep teeth, but I can never get what I want. I buy *my* teeth from Jones, White & McCurdy's Agent, $50 worth at a time, and go there only when I want a tooth that I *can not find* in *my own stock*." Comment is unnecessary. Who would be *fool* enough to keep a large stock of Goods to accommodate such customers!

The Depots in Cincinnati would gladly sell teeth in the *same quantities*, at as liberal prices as you pay East.

EASTERN MANUFACTURERS, also, pursue a course of doubtful policy; they appoint local agents to sell their teeth, and sell in large quantities to *dealers*—then send out traveling agents through the country to forestall the trade, and if possible, cut off every chance of the local dealer, to dispose of the goods they have sold him, thus destroying that energy and enterprise in local dealers which would be productive of *greater* benefits to all parties.

